# Genetic variants in the fat mass and obesity-associated (*FTO*) gene confer risk for extreme obesity and modulate adiposity in a Brazilian population

**DOI:** 10.1590/1678-4685-GMB-2018-0264

**Published:** 2020-03-06

**Authors:** Ana Carolina Proença da Fonseca, Bruna Marchesini, Verônica Marques Zembrzuski, Danielle Dutra Voigt, Vivianne Galante Ramos, João Regis Ivar Carneiro, José Firmino Nogueira, Giselda Maria Kalil de Cabello, Pedro Hernán Cabello

**Affiliations:** 1 Instituto Oswaldo Cruz (FIOCRUZ), Laboratório de Genética Humana, Rio de Janeiro, RJ, Brazil.; 2 Universidade do Grande Rio, Laboratório de Genética Humana, Rio de Janeiro, RJ, Brazil.; 3 Universidade Federal do Rio de Janeiro, Hospital Universitário Clementino Fraga Filho, Rio de Janeiro, RJ, Brazil.; 4 Universidade do Estado do Rio de Janeiro, Departamento de Patologia, Rio de Janeiro, RJ, Brazil.

**Keywords:** BMI, extreme obesity, FTO, haplotype, polymorphisms

## Abstract

Obesity is a major public health problem worldwide. It has a complex etiology, influenced by environmental and genetic factors. *FTO* has been recognized as an important genetic factor for obesity development. This study evaluated the contribution of *FTO* polymorphisms (rs9939609 and rs17817449) for extreme obesity in terms of the period of obesity onset, anthropometric, and biochemical parameters. The haplotype and the combined effects of *FTO* risk alleles on obesity susceptibility were evaluated. We investigated 169 normal-weight subjects (body mass index, BMI: 22.8 [21.0; 24.0] kg/m^2^) and 123 extremely obese individuals (BMI: 47.6 [44.1; 53.1] kg/m^2^). Genotyping was performed by real time PCR. Our results showed a strong association between *FTO* variants and extreme obesity. Carriers of the AT haplotype had an increased risk for extreme obesity. Gene scores suggested that the risk of developing extreme obesity was increased 1.37-fold per risk allele added. Both polymorphisms also influenced BMI and body weight. Additionally, rs17817449 influenced triglyceride levels. No effect of *FTO* variants on the period of obesity onset was found. In conclusion, the *FTO* polymorphisms showed a strong association with development of extreme phenotype of obesity and adiposity modulation in a Brazilian population.

## Introduction

Obesity (body mass index, BMI ≥ 30 kg/m^2^) is defined as an increase in body fat mass that is sufficient to cause adverse health effects. Over the last three decades, the prevalence of obesity has increased rapidly, affecting low-, middle-, and high-income countries. Recent data estimate that there are at least 600 million obese people in the world. In Brazil, 20% of the population was obese in 2015 (WHO, 2018). Epidemiological studies have shown that subjects with severe obesity and morbid obesity (BMI ≥ 35 kg/m^2^ and BMI ≥ 40 kg/m^2^, respectively) have a substantial increase in the risk of comorbidities and mortality. Furthermore, this elevated BMI was associated with 6.5–13.7 years lost in life expectancy (Kitahara *et al.*, 2014). The prevalence of severe obesity in women is three times higher than men in Brazil. There are 6.7 million women with this disease, whereas only 2.2 million men. Currently, the prevalence of morbid obesity is nearly 1.1% in the world (NCD Risk Factor Collaboration, 2016).

Obesity is a multifactorial disease, influenced by environmental and genetic factors (Grundy, 1998). Researchers have been making a continuous effort to identify genes and variants that predispose individuals to common forms of obesity (Rankinen *et al.*, 2006; Fonseca *et al.*, 2017). Nowadays, over 291 loci were found associated with obesity, involved in different biological pathways, including adipocyte differentiation, lipid metabolism, thermogenesis, and food intake control (Wu *et al.*, 2018). Among the genes, the association of fat mass and obesity-associated gene (*FTO*) with obesity susceptibility was confirmed. Different groups identified that common variants in the first intron of *FTO* are associated with an increased body fat mass in humans (Dina *et al.*, 2007; Frayling *et al.*, 2007; Scuteri *et al.*, 2007). Besides the association with obesity, *FTO* variants have also been correlated to body composition and obesity-related traits (Scuteri *et al.*, 2007; Shabana and Hasnain, 2015).

*FTO* encodes a Fe(II)- and 2-oxoglutarate (OG)-dependent nucleic acid demethylase that is localized in the cell nucleus. The highest levels of *FTO* mRNA are found in the hypothalamus, particularly in the arcuate nucleus. This area of the brain has an important role in controlling food intake, which suggests that *FTO* may act in the management of energy balance (Gerken *et al.*, 2007). Located in chromosome 16, the *FTO* gene spans for more than 400 kb and has nine exons. Despite the large size of this gene, most of the variations that were associated with obesity are found in the first intron (Loos and Bouchard, 2008).

The influence of *FTO* variants on the risk of obesity is consistent in Caucasian studies (Dina *et al.*, 2007). However, apparently it is not relevant in African Americans, Chinese Han, and Native Oceanic populations (Ohashi *et al.*, 2007; Scuteri *et al.*, 2007; Li *et al.*, 2008). In Brazil, few studies have analyzed the relationship between *FTO* rs9939609 polymorphism and obesity susceptibility. Reuter *et al.* (2016) studied the association between this polymorphism and overweight/obesity risk in a sample of youths from the South of Brazil. They observed that the rs9939609 was associated with BMI and waist circumference. Pereira *et al.* (2016) have performed a similar study with children; however, they did not find this association. Ramos *et al.* (2012) investigated the contribution of this polymorphism in morbid obesity risk. They analyzed 126 morbidly obese individuals and 113 controls from Minas Gerais, in the southeastern region of Brazil. Their result suggested that presence of the rs9939609(A) allele increased the risk for obesity. These divergent results may be explained by differences in the inclusion criteria of participants, as well as environmental and genetic backgrounds. Therefore, more studies are needed to elucidate the association of *FTO* rs9939609 and obesity development in Brazil. In addition, we also selected the *FTO* rs17817449 polymorphism for analysis. To date, no study was performed with this polymorphism in the Brazilian population.

The aim of our study was to evaluate the role of the *FTO* rs9939609 and rs17817449 polymorphisms in the risk of extreme obesity, period of obesity onset, and in relation to anthropometric and biochemical parameters. We also investigated the influence of haplotype and the combined effect of *FTO* variants on extreme obesity development.

## Materials and Methods

### Subjects

This cross-sectional case-control study comprised 292 unrelated individuals (68.2% female and 31.8% male), aged 18 to 65 years (median, 33.0 [26.0; 43.0]), from Rio de Janeiro, southeast of Brazil. The participants were divided into two groups according to BMI (calculated by dividing weight [kilograms] by the height squared [meters]). The exclusion criteria were pregnancy, lactation, and the use of medication to lose or gain weight. The first group included 123 subjects with extreme obesity (BMI ≥ 40.0 kg/m^2^) recruited from a non-governmental organization called to Self-Esteem and Citizenship Rescue Group for the Obese (in Portuguese, “*Grupo de Resgate à Autoestima e Cidadania do Obeso*”). These patients were candidates for bariatric surgery. The period of obesity onset was self reported. The second group included 169 individuals with normal weight (18.5 ≤ BMI ≤ 24.9 kg/m^2^) that were volunteers in public hospitals in the same city. The study protocol was performed according to the Declaration of Helsinki (1964). All participants provided written consent prior to their inclusion in this study and the protocol was approved by Ethics Committee of the Oswaldo Cruz Foundation.

### Anthropometric measurements

Height, weight, and waist and hip circumference were measured by a trained person. Waist circumference was measured at the midpoint between the iliac crest and the last costal arch. Hip circumference was measured at the level of the greater trochanters. BMI and waist to hip ratio (WHR) were then calculated for each subject.

### Biochemistry

Serum samples were collected and clinical variables were measured after an overnight fast. Glucose, total cholesterol (TC), HDL-cholesterol (HDL-c), and triglyceride (TG) were measured by the oxidase-peroxidase method (BioSystems). LDL-cholesterol (LDL-c) was calculated by the Friedewald formula (LDL-c = TC – HDL-c – TG/5). Individuals using medication for these biochemical parameters had their levels excluded from statistical analysis.

### Demographic characteristics

Practice of physical activity was categorized as “yes” or “no” according to the participant’s report of the last month. The classification of the Demographic Census conducted by the Brazilian Institute of Geography and Statistics was used to categorize the race/skin color of participants (white, brown, black, and others [yellow or indigenous]). All information was self reported and collected using standardized questionnaires.

### Single nucleotide polymorphism (SNP) genotyping

Genomic DNA was extracted from peripheral white blood cells using a commercial DNA extraction kit (QIAamp Blood Kit, Qiagen, Valencia, CA, USA). *FTO* rs9939609 and rs17817449 polymorphisms were genotyped by real time PCR using TaqMan^®^ assays (ThermoFisher, Carlsbad, CA, USA). Reactions were performed in 10 μL volumes, including 2–10 ng of DNA, Universal Master Mix 1X, and TaqMan Genotyping Assay 1X specific for each polymorphism studied. Amplification was carried out in a StepOne^®^ Plus Real Time PCR System (ThermoFisher) using the number of cycles and temperatures according the manufacturer’s recommendations. All plates included a negative control (all components excluding DNA).

### Statistical analysis

Normality of continuous variables was tested by Kolmogorov-Smirnov and Shapiro Wilk tests. All continuous parameters had a non-normal distribution. Differences of clinical, biochemical, and anthropometric parameters between case and control groups were compared by Mann Whitney and χ^2^ tests.

Genotype and allele frequencies were estimated by gene counting. Hardy-Weinberg equilibrium (HWE) was tested for each polymorphism using the χ^2^ test. Logistic regression was carried out in order to analyze the association between *FTO* polymorphisms and the risk for extreme obesity. All analyses were adjusted for age and gender (model 1) and then for race/skin color and physical activity practice (model 2). Anthropometric and biochemical variables were log-transformed before linear regression. All linear regression analyses were adjusted by model 1 and 2. We also included BMI as covariate, excepted for BMI and body weight. Both logistic and linear regressions were carried out in additive, recessive, and dominant models.

Haplotypes were obtained and their associations with obesity were verified by χ^2^ test (OR was calculated). A pairwise linkage disequilibrium analysis between rs9939609 and rs17817449 polymorphisms was carried out. Furthermore, gene score + was used to calculate the combined risk alleles. Individuals were encoded as 0 (homozygous protective), 1 (heterozygous), and 2 (homozygous risk) for each variant studied (Shabana and Hasnain, 2015; Shabana *et al.*, 2016). Then, the number of risk alleles was calculated from the sum of the codes for both rs9939609 and rs17817449 polymorphisms. The mean of risk alleles was compared between the groups using Student’s *t*-test*.* Logistic regression was also used to calculate the extreme obesity risk per unit allele increased. Finally, period of obesity onset was obtained from individuals with extreme obesity phenotype. The Mann-Whitney test was used to calculate BMI differences between subjects with early and late-onset obesity. Finally, the association of *FTO* polymorphisms with the period of obesity onset was tested using logistic regression. Since different polymorphisms were analyzed, a Bonferroni’s correction was used and a *p*-value of 0.025 was applied as a significant cutoff. All data analyses were performed using the SPSS statistical package, Haploview, and PHASE.

Sample size calculation was performed using an iterative process to compute the minimum number of individuals necessary to test the difference between two groups of a qualitative variable according to Zar (1999). In this study, rs9939609 and rs17817449 polymorphisms were analyzed, and thus a conservative and convenience sample was chosen (80% of statistic power).

## Results

### Basic characteristics of the study population

Clinical characteristics from the 292 subjects, stratified into case and controls by BMI status, are shown in Table 1. As expected, anthropometric and biochemical data were significantly increased in individuals with extreme obesity when compared to the control group. Exceptions were in HDL-cholesterol and height, in which the control individuals had higher values. We also observed that the control group practiced more physical activities and exhibited a higher proportion of subjects with white race/skin color.

**Table 1:** Characteristics of study population.

Variables	N	All	n	Control	n	Case	*p*
Age (years)	292	33 (26; 43)	169	29 (24; 38)	123	39 (31; 49)	<**0.001**
Gender (female/male)	292	199/93	169	100/69	123	99/24	<**0.001**
Race/ Skin color							
White	291	163 (56.0)	169	114 (67.5)	122	49 (40.2)	<**0.001**
Brown		84 (28.9)		44 (23)		40 (32.8)	
Black		42 (14.4)		11 (6.5)		31 (25.4)	
Others		2 (0.7)		0 (0)		2 (1.6)	
Physical Activity Practice							
Yes	289	116 (40.1)	169	88 (52.1)	120	28 (23.3)	<**0.001**
No		173 (59.9)		81 (47.9)		92 (76.7)	
Weight (kg)	292	74.1 (61.3; 126.2)	169	63.0 (57.0; 70.3)	123	131.5 (115.5; 146.5)	<**0.001**
Height (m)	292	1.65 (1.60; 1.73)	169	1.69 (1.62; 1.74)	123	1.62 (1.58; 1.69)	<**0.001**
BMI (kg/m^2^)	292	24.6 (22.4; 46.61)	169	22.8 (21.0; 24.0)	123	47.6 (44.1; 53.1)	<**0.001**
Waist circumference (cm)	292	98.0 (83.0; 134.0)	169	84.0 (76.0; 91.0)	122	138.3 (128.0; 148.3)	<**0.001**
Hip circumference (cm)	292	103.0 (92.5; 141.0)	169	95.0 (85.3; 100.3)	122	144.0 (134.0; 154.3)	<**0.001**
WHR	292	0.92 (0.83; 1.01)	169	0.86 (0.80; 1.04)	122	0.97 (0.91; 1.01)	<**0.001**
Glucose (mg/dL)	259	92.0 (86.0; 102 5)	163	88.0 (84.0; 95.0)	96	103.0 (94.0; 115.8)	<**0.001**
Total cholesterol (mg/dL)	259	182.0 (159.0; 209 0)	163	178.0 (156.0; 198.0)	96	192.0 (168.0; 220.8)	**0.003**
HDL cholesterol (mg/dL)	259	52.0 (44.0; 63.0)	163	58.0 (47.0; 68.0)	96	47.0 (41.0; 53.8)	<**0.001**
LDL cholesterol (mg/dL)	255	108.0 (88.0; 127 0)	162	102.0 (86.8; 122.3)	93	116.0 (95.5; 137.0)	**0.002**
Triglycerides (mg/dL)	259	88.0 (68.0; 131.0)	163	76.0 (61.0; 102.0)	96	128.0 (93.8; 185.0)	<**0.001**

Data are presented as medians values (interquartile range) for continuous traits and n (%) for categorical traits. Data were analyzed by Mann-Whitney Test (for non-normally distributed variables), or χ^2^ test (for categorical variables).

BMI, Body Mass Index; WHR, Waist-to-Hip Ratio; HDL cholesterol, High Density Lipoprotein-cholesterol; LDL cholesterol, Low Density Lipoprotein-cholesterol.

*p-*value for difference between case and control groups.

### FTO frequencies and Hardy-Weinberg equilibrium

All subjects were genotyped for *FTO* rs9939609 and rs17817449 variants. Both were polymorphic in our sample. The details about the genotypic and allelic frequencies are presented in Table 2. Genotypes of rs9939609 and rs17817449 polymorphisms were in HWE (*p* > 0.05) for both case and control groups.

**Table 2:** Genotypic and allelic frequencies of *FTO* rs9939609 and rs17817449 polymorphisms.

*FTO* polymorphisms	Control	Case	Model 1		Model 2	
	n=169 (%)	n=123 (%)	OR (95% CI)	*P*	OR (95% CI)	*p*
**rs9939609**						
*Genotype*						
TT	64 (37.9)	27 (22.0)	1.00 (Ref.)	-	1.00 (Ref.)	-
TA	72 (42.6)	59 (48.0)	1.97 (1.06 - 3.66)	0.032	1.90 (0.99 - 3.66)	0.054
AA	33 (19.5)	37 (30.1)	2.88 (1.41 - 5.89)	**0.004**	2.51 (1.17 - 5.35)	**0.017**
*Dominant Model*						
TT	64 (37.9)	27 (22.0)	1.00 (Ref.)	-	1.00 (Ref.)	-
TA+AA	105 (62.1)	96 (78.0)	2.25 (1.26 - 4.01)	**0.006**	2.09 (1.14 - 3.84)	**0.016**
*Recessive Model*						
TT+TA	136 (80.5)	86 (69.9)	1.00 (Ref.)	-	1.00 (Ref.)	-
AA	33 (19.5)	37 (30.1)	1.91 (1.05 - 3.48)	0.035	1.74 (0.90 - 3.33)	0.097
*Allele*						
T	200 (59.2)	113 (45.9)	1.00 (Ref.)	-	1.00 (Ref.)	-
A	138 (40.8)	133 (54.1)	1.70 (1.19 - 2.43)	**0.003**	1.60 (1.10 - 2.33)	**0.014**
**rs17817449**						
*Genotype*						
G/G	64 (37.9)	33 (26.8)	1.00 (Ref.)	-	1.00 (Ref.)	-
G/T	91 (53.8)	60 (48.8)	1.41 (0.78-2.52)	0.365	1.21 (0.65-2.26)	0.531
T/T	14 (8.3)	30 (24.4)	4.74 (2.04 - 11.01)	**0.0003**	3.89 (1.56 – 9.38)	**0.003**
*Dominant Model*						
GG	64 (37.9)	33 (26.8)	1.00 (Ref.)	-	1.00 (Ref.)	-
GT+TT	105 (62.1)	90 (73.2)	1.83 (1.05 - 3.19)	0.034	1.57 (0.87 - 2.82)	0.130
*Recessive Model*						
GG+GT	155 (91.7)	93 (75.6)	1.00 (Ref.)	-	1.00 (Ref.)	-
TT	14 (8.3)	30 (24.4)	3.83 (1.80 - 8.17)	**0.001**	3.41 (1.50 - 7.74)	**0.003**
*Allele*						
G	219 (64.8)	126 (51.2)	1.00 (Ref.)	-	1.00 (Ref.)	-
T	119 (35.2)	120 (48.8)	1.98 (1.33 - 2.96)	**0.001**	1.75 (1.15 - 2.67)	**0.009**

Model 1 adjusted for gender and age.

Model 2 adjusted for gender, age, race/skin color and physical activity practice.

### Influence of FTO variants on extreme obesity risk

The distribution of genotypes and alleles for rs9939609 and rs17817449 polymorphisms was compared between case and control groups (Table 2). Our results showed that the genotypic and allelic frequencies of both polymorphisms differed significantly between the groups. The frequency of the rs9939609(AA) genotype was significantly higher in the extremely obese group when compared to the control group. Allelic analysis showed that the prevalence of the rs9939609(A) allele was higher in subjects with extreme obesity (0.54 vs. 0.41; *p* = 0.003). Individuals carrying the rs9939609(A) allele had a 1.7 times higher risk for developing this phenotype.

The frequency of the rs17817449(TT) genotype was higher in the case than in the control group. Furthermore, subjects carrying the TT genotype had a 4.5-fold increased risk for extreme obesity. In addition, carriers of at least one rs17817449(T) allele were 1.98 times more likely to develop this extreme phenotype. All these associations remained after adjusting for covariates (model 2).

### Haplotype and linkage disequilibrium

Haplotype group analyses were performed using *FTO* rs9939609 and rs17817449. In the whole sample, the most common haplotypes were TG (50%) and AT (39.5%). The distribution of haplotypes between the case and control groups is shown in Table 3. Our results demonstrated that carriers of the AT haplotype had a 1.87-fold increased risk for extreme obesity development when compared to individuals with TG haplotype.

**Table 3:** Association of *FTO* haplotypes with obesity susceptibility.

Haplotype	Control	Extremely obese	OR (95% CI)	*p*
	n=169 (%)	n=123 (%)		
T/G	187 (55.3)	110 (44.7)	1.00 (Ref.)	-
T/T	13 (3.8)	3 (1.2)	0.44 (0.14 - 1.40)	0.156
A/G	32 (9.5)	16 (6.5)	0.86 (0.46 - 1.62)	0.639
A/T	106 (31.4)	117 (47.6)	1.87 (1.32 - 2.66)	**0.0005**

The polymorphisms studied are located in the first intron of *FTO,* and the distance from one another is about 7.2 kb. Our analysis demonstrated that *FTO* rs9939609 and rs17817449 are in linkage disequilibrium (D’ = 0.78; r^2^ = 0.583; LOD = 52.63).

### Additive effects of FTO risk alleles on obesity

The combined effect of risk alleles was performed in order to study their influence on extreme obesity development. Our results showed that the average number of risk alleles was different between the groups (cases = 2.05 ± 1.34; controls = 1.52 ± 1.34; *p* = 0.001). Furthermore, the risk of being obese increased 1.37-fold for each risk allele added (OR = 1.37 [1.14 – 1.66]; *p* = 0.001).

### Association of FTO variants with biochemical and anthropometric parameters

The influence of both *FTO* polymorphisms on biochemical and anthropometric measurements was analyzed by linear regression. *FTO* rs9939609 was associated with body weight in the additive model. Moreover, it was associated with BMI in the additive, dominant, and recessive models (Table 4). However, the association with BMI in the recessive model did not remain after correcting for covariates (model 2). *FTO* rs17817449 was associated with body weight in the additive and recessive models. This variant was also related with BMI in the additive, dominant, and recessive models. Furthermore, *FTO* rs17817449 influenced the triglycerides levels in the recessive model (Table 5). Our results indicated no significant effects of rs9939609 and rs17817449 variants on fasting glucose levels, total cholesterol, HDL-cholesterol, LDL-cholesterol, WHR, and waist and hip circumferences.

**Table 4:** Association of *FTO* rs9939609 with anthropometric and biochemical parameters.

Parameters	TT		TA		AA		Additive		Dominant		Recessive	
	n	Values	N	Values	n	Values	*p* ^*a*^	*p* ^b^	*p* ^*a*^	*p* ^b^	*p* ^*a*^	*p* ^b^
Weight (kg)	91	69.5 (57.2 - 81.2)	131	70.5 (59.0 - 117.6)	70	70.0 (60.1 - 126.3)	**0.008**	**0.021**	0.028	0.041	0.027	0.079
BMI (kg/m^2^)	91	23.4 (21.2 - 24.9)	131	24.1 (21.8 - 44.9)	70	24.5 (22.9 - 46.0)	**0.003**	**0.009**	**0.011**	**0.016**	**0.017**	0.055
Waist circumference (cm)	91	87.5 (82.0 - 102.5)	130	96.5 (81.8 - 128.1)	70	91.0 (80.0 - 126.5)	0.996	0.813	0.597	0.703	0.561	0.409
Hip circumference (cm)	91	98.5 (90.0 - 106.75)	130	102.5 (88.0 - 136.8)	70	99.5 (91.0 - 138.5)	0.234	0.207	0.697	0.636	0.103	0.095
WHR	91	0.91 (0.82 - 1.04)	130	0.92 (0.82 - 1.03)	70	0.90 (0.83 - 1.04)	0.252	0.255	0.576	0.556	0.172	0.184
Glucose (mg/dL)	76	97.0 (88.0 - 116.8)	105	96.0 (88.0 - 109.3)	46	99.0 (90.5 - 113.5)	0.828	0.850	0.938	0.933	0.633	0.663
Total cholesterol (mg/dL)	82	180.5 (154.0 - 199.3)	119	183.5 (16.8 - 200.3)	58	180.0 (155.0 - 210.0)	0.063	0.073	0.090	0.104	0.179	0.191
HDL cholesterol (mg/dL)	82	54.0 (45.3 - 63.0)	119	52.0 (43.0 - 63.3)	58	53.0 (44.5 - 67.5)	0.085	0.110	0.369	0.365	0.078	0.077
LDL cholesterol (mg/dL)	82	106.5 (84.3 - 126.0)	117	109.0 (89.8 - 129.3)	56	102.0 (86.5 - 125.0)	0.240	0.261	0.221	0.245	0.496	0.509
Triglycerides (mg/dL)	82	83.5 (66.0 - 125.8)	119	86.0 (65.5 - 120.3)	58	85.0 (67.5 - 127.5)	0.460	0.551	0.838	0.699	0.130	0.142

Data are present as median values (interquartile range) for genetic classes based on unrelated individuals. Dominant indicates TT and TA vs AA for rs9939609. Recessive indicates TT vs TA and AA for rs9939609. Abbreviations: BMI, Body Mass Index; WHR, Waist-to-Hip Ratio; HDL, High Density Lipoprotein-cholesterol; LDL, Low Density Lipoprotein-cholesterol. *p*^a^-value for linear regression. Weight and BMI were adjusted by gender and age; other parameters were adjusted by gender, age and BMI. *p*^b^- value for linear regression. Weight and BMI were adjusted by gender, age, race/skin color and physical exercise practice; other parameters were adjusted by gender, age, race/skin color, physical exercise practice and BMI.

**Table 5:** Association of *FTO* rs17817449 with anthropometric and biochemical parameters.

Parameters	GG		GT		TT		Additive		Dominant		Recessive	
	n	Values	N	Values	n	Values	*p* ^*a*^	*p* ^b^	*p* ^*a*^	*p* ^b^	*p* ^*a*^	*p* ^b^
Weight (kg)	97	69.5 (57.4 - 80.9)	151	70.5 (59.0 - 117.3)	44	70.0 (60.2 - 124.8)	**0.0005**	**0.003**	0.032	0.077	**0.0003**	**0.001**
BMI (kg/m2)	97	23.4 (21.3 - 24.9)	151	24.1 (21.9 - 44.8)	44	24.5 (22.9 - 42.4)	**0.0003**	**0.002**	**0.017**	0.041	**0.0003**	**0.002**
Waist circumference (cm)	97	87.5 (82.0 - 102.0)	150	96.5 (82.0 - 128.0)	44	91.0 (80.0 - 126.0)	0.712	0.913	0.624	0.777	0.970	0.859
Hip circumference (cm)	97	98.5 (90.0 - 106.5)	150	102.5 (88.0 - 136.0)	44	99.5 (92.0 - 136.0)	0.340	0.315	0.787	0.777	0.151	0.127
WHR	97	0.91 (0.82 - 1.04)	150	0.92 (0.82 - 1.03)	44	0.90 (0.83 - 1.03)	0.296	0.313	0.663	0.705	0.167	0.161
Glucose (mg/dL)	80	97.0 (88.0 - 116.5)	124	96.0 (88.0 - 109.0)	23	99.0 (92.0 - 113.0)	0.664	0.709	0.636	0.670	0.861	0.926
Total cholesterol (mg/dL)	86	180.5 (154.0- 198.5)	138	183.5 (161.0 - 200.0)	35	180.0 (156.0 - 209.0)	0.424	0.408	0.735	0.720	0.288	0.278
HDL cholesterol (mg/dL)	86	54.0 (45.5 - 63.0)	138	52.0 (43.0 - 63.0)	35	53.0 (45.0 - 67.0)	0.808	0.811	0.518	0.522	0.168	0.173
LDL cholesterol (mg/dL)	86	106.5 (84.5 - 126.0)	136	109.0 (90.0 - 129.0)	33	102.0 (87.0 - 124.0)	0.990	0.950	0.969	0.950	0.975	0.975
Triglycerides (mg/dL)	86	83.5 (67.0 - 125.5)	138	86.0 (66.0 - 120.0)	35	85.0 (68.0 - 125.0)	0.145	0.122	0.851	0.813	**0.011**	**0.008**

Data are present as median values (interquartile range) for genetic classes based on unrelated individuals. Dominant indicates GG and GT vs TT for rs17817449. Recessive indicates GG vs GT and TT for rs17817449. Abbreviations: BMI, Body Mass Index; WHR, Waist-to-Hip Ratio; HDL, High Density Lipoprotein-cholesterol; LDL, Low Density Lipoprotein-cholesterol. *p*^*a*^ -value for linear regression. Weight and BMI were adjusted by gender and age; other parameters were adjusted by gender, age and BMI. *p*^b^- value for linear regression. Weight and BMI were adjusted by gender, age, race/skin color and physical exercise practice; other parameters were adjusted by gender, age, race/skin color, physical exercise practice and BMI.

### Association of FTO variants with onset of obesity

We divided the case group according to the period of obesity onset, stratifying in early-onset obesity (childhood and adolescence) and late-onset obesity (adulthood). Of the 123 individuals with extreme obesity, 70 subjects developed obesity during childhood/adolescence (56.9%), and 53 individuals developed obesity during adulthood (43.1%). Our results showed no relation between BMI and period of obesity onset, in which both early-onset (BMI = 48.84 [44.65; 55.05] kg/m^2^) and late-onset (BMI = 46.92 [43.11; 52.87] kg/m^2^) had similar median values (*p* = 0.376). We also analyzed whether those polymorphisms influence the period of obesity onset. However, no association was found for both variants (data not shown).

## Discussion

Obesity is a complex phenotype influenced by genetic and environmental factors. In the last decades, individuals have changed the lifestyle, resulting in energy imbalance caused by excessive food intake and diminished physical activity. Regarding genetic factors, the *FTO* gene has been recognized as one of the main contributors to polygenic obesity. However, the influence of *FTO* variants has been controversial among different populations (Ohashi *et al.*, 2007; Scuteri *et al.*, 2007; Li *et al.*, 2008; Ramos *et al.*, 2012; Pereira *et al.*, 2016). Moreover, there are only few studies associating *FTO* variants and extreme obesity, since sample selection is difficult and requires considerable effort. The strategy of using an extreme phenotype sample probably increases the power for detecting genetic associations in a median sample size (Price *et al.*, 2008; Castro *et al.*, 2015).

In this study, we observed a strong association between the *FTO* rs9939609 and rs17817449 polymorphisms and risk for extreme obesity in a Brazilian population sample. These polymorphisms are located in the first intron of *FTO*, which is a region that has been consistently associated with adiposity in humans (Dina *et al.*, 2007; Frayling *et al.*, 2007; Scuteri *et al.*, 2007). Interestingly, the rs9939609 and rs17817449 polymorphisms were associated with extreme obesity in different populations (Price *et al.*, 2008; Villalobos-Comparán *et al.*, 2008). Price *et al.* (2008) found that the rs9939609(A) and rs17817449(G) alleles were risk factors for extreme obesity in women from Spain. However, González *et al.* (2012) reported that only rs17817449(T) was associated with extreme obesity in individuals from Western Spain when a single analysis was performed. Ramos *et al.* (2012) observed that presence of the rs9939609(A) allele increased the risk for morbid obesity in a Brazilian population. Furthermore, Villalobos-Comparán *et al.* (2008) reported that both *FTO* variants were in strong linkage disequilibrium and were associated with extreme obesity in Mexican mestizos subjects, which was corroborated by our results.

Different studies have analyzed the contribution of single nucleotide polymorphisms for obesity risk. However, common forms of obesity have a complex etiology and could be explained by combined effects of variants located in the same or different genes (Shabana and Hasnain, 2015). Our study showed that the mean values of *FTO* risk alleles were higher in the case group. We also found that the risk of developing extreme obesity increased for each risk allele added. Only few previous studies had performed such an analysis. Nevertheless, it is a robust approach when the genetic study has a small or median sample size (Rouskas *et al.*, 2012; Lazopoulou *et al.*, 2015; Shabana and Hasnain, 2015). Shabana and Hasnain (2015) performed this analysis using the rs9939609 polymorphism and other different variants in the same gene. Interestingly, they reported that the mean of risk alleles was elevated in an obese group from Pakistan. Our results are in accordance with earlier studies that reported that the homozygote risk variants for both polymorphisms are strongly associated with obesity susceptibility (Price *et al.*, 2008; Villalobos-Comparán *et al.*, 2008).

We also tested the influence of these polymorphisms on anthropometric and biochemical parameters. Our results showed that rs9939609 and rs17817449 were associated with BMI and body weight. Furthermore, the rs17817449 polymorphism was also associated with triglycerides levels. A variety of studies has investigated the effect of rs9939609 and rs17817449 variants on metabolic and anthropometric measurements, and these reported divergent results (Sentinelli *et al.*, 2012; Lazopoulou *et al.*, 2015; Shabana *et al.*, 2016). These different results may be explained by the effect of these two *FTO* variants that depend on environmental factors and ethnicity of the population.

Additionally, we investigated the influence of *FTO* variants on the period of obesity onset. No association was found in our population. Previous studies have reported that the rs9939609 polymorphism was associated with body weight according to age. Sentinelli *et al.* (2012) suggested that individuals carrying the rs9939609(A) risk allele had an increased body size earlier in life. Another study investigated the longitudinal pattern of the relationship between the rs9939609 variant and BMI during childhood, adolescence, and adulthood. This polymorphism was strongly associated with increased BMI during childhood, adolescence, and early adulthood (Hardy *et al.*, 2010). Reasons for the discrepancies in these results may be explained by a different methodology design, since we have performed a cross-sectional study. Furthermore, the period of obesity onset was self reported in our study, whereas Sentinelli *et al.* (2012) used the age of individuals at the moment of sample collection.

The role of FTO protein in obesity is not completely elucidated. However, high levels of FTO mRNA were detected in a hypothalamus region that is involved in energy homeostasis. Animal studies suggested that *Fto* expression is regulated by feeding and fasting (Gerken *et al.*, 2007). Church *et al.* (2010) also reported that loss of function or expression of *Fto* is associated with an increased energy expenditure and a lean phenotype; otherwise, the overexpression induced an increased food intake and resulted in obesity.

Even though the two polymorphisms are not located in an encoding region, they may exert functional effects through altered levels of FTO mRNA (Gerken *et al.*, 2007), or they are in linkage disequilibrium with another causative genetic variant (Prakash *et al.*, 2016). Additionally it has been suggested that the polymorphisms located in noncoding sequences (intron 1 and 2) within *FTO* interact with the promotor region of another gene in the neighborhood, called *IRX3*. Consistent with this, *FTO* polymorphisms associated with obesity alter the expression of IRX3 in human brains, which is related with regulation of body mass and composition (Smemo *et al.*, 2014). Therefore, our results suggest that *FTO* rs9939609 and rs17817449 are risk factors for obesity; however, more functional studies are required to confirm these results. 

## Conclusion

In this study, we investigated the association between *FTO* polymorphisms (rs9939609 and rs17817449) and the risk for extreme obesity development. We identified that *FTO* rs9939609 and rs17817449 polymorphisms have a strong association with extreme obesity and adiposity modulation in a Brazilian population sample.
